# Clinico-Etiological Spectrum and Outcome in Patients With Septic Acute Kidney Injury and Its Comparison With Non-septic Acute Kidney Injury: A Hospital-Based Prospective Study Conducted in a Tertiary Care Hospital in North India

**DOI:** 10.7759/cureus.37857

**Published:** 2023-04-19

**Authors:** Sheikh Tahir, Basharat Ahmed Ganie, Touseef Yaqoob Beigh, Aqib Jalal Hazar, AR Reshi

**Affiliations:** 1 Geriatrics, Sher-I-Kashmir Institute of Medical Sciences (SKIMS), Srinagar, IND; 2 Internal Medicine, Sher-I-Kashmir Institute of Medical Sciences (SKIMS), Srinagar, IND; 3 Nephrology, Sher-I-Kashmir Institute of Medical Sciences (SKIMS), Srinagar, IND

**Keywords:** etiology, nephrotoxin, mortality, sepsis, aki

## Abstract

Background

Acute kidney injury (AKI) is a heterogenous syndrome defined by the impairment of kidney filtration and excretory function over days to weeks, resulting in the retention of nitrogenous and other waste products normally cleared by the kidneys. In addition, AKI is frequently recognized to be associated with sepsis and contributes to an unfavorable outcome in sepsis. This study was undertaken to study and compare the etiology and clinical profile of patients with septic and non-septic AKI and to study and compare the outcome in both groups.

Materials and methods

This is a prospective, observational, and comparative study with a total sample size of 200 patients selected randomly having sustained an acute kidney injury. Data was collected, recorded, analyzed, and compared for two groups of patients with septic and non-septic AKI.

Results

A total of 200 cases of AKI were enrolled, out of which 120 (60%) were due to non-septic etiology and 80 (40%) were of septic etiology. Urosepsis (37.5%) due to various urinary tract infections including pyelonephritis and chest sepsis (18.75%) including community-acquired pneumonia (CAP) and aspiration pneumonia were the predominant causes of sepsis. AKI secondary to nephrotoxic agents (27.5%) was the commonest cause in the non-septic group, followed by glomerulonephritis (13.3%), vitamin D intoxication-related hypercalcemia (12.5%), acute gastroenteritis (10.8%), etc. Patients of septic AKI had a significantly higher mean of maximum urea and creatinine than their non-septic AKI counterparts. In addition to having an increased duration of hospital stay, mortality was significantly higher in patients with septic AKI (27.5%) than in patients with non-septic AKI (4.1%). However, sepsis had no effect on renal functions, measured by urea and creatinine, at discharge. In patients with AKI, certain factors were found to increase the risk of mortality. These factors include being over 65 years old, needing mechanical ventilation or vasopressors, requiring renal replacement therapy (RRT), and having multiorgan dysfunction syndrome (MODS), septic shock, or acute coronary syndrome (ACS). However, pre-existing conditions such as diabetes, hypertension, malignancy, previous stroke, chronic kidney disease (CKD), and chronic liver disease (CLD) did not affect the overall mortality risk.

Conclusion

In the septic AKI group, urosepsis was the most frequent etiology of AKI, while the most frequent etiology of AKI in the non-septic group was nephrotoxin exposure. Patients with septic AKI had considerably longer hospital stays and greater in-hospital mortality rates than patients with non-septic AKI. The renal functions as determined by urea and creatinine at discharge were unaffected by sepsis. Finally, death was significantly impacted by age of >65 years, the necessity for mechanical ventilation, the use of vasopressors and RRT, and the presence of MODS, septic shock, and ACS.

## Introduction

Acute kidney injury (AKI) has now replaced the term acute renal failure to consider the disease as a spectrum of injury extending from less severe forms to more advanced injury when renal replacement therapy (RRT) may be required [1]. A diagnosis of AKI is currently made on the basis of the presence of increased serum creatinine and/or blood urea nitrogen (BUN) levels and/or decreased urine output, despite their well-known limitations.

AKI is usually divided into three broad pathophysiologic categories based on cause: 1) prerenal AKI, diseases characterized by effective hypoperfusion of the kidneys in which there is no parenchymal damage to the kidney [2]; 2) intrinsic AKI, diseases involving the renal parenchyma [2]; and 3) postrenal (obstructive) AKI, diseases associated with acute obstruction of the urinary tract [2]. Elderly patients are susceptible to many forms of acute renal failure because the aging kidney loses functional reserve and its ability to withstand acute insults is compromised [3]. AKI occurs in approximately 19% of patients with moderate sepsis, 23% of patients with severe sepsis, and 51% of patients with septic shock [4]. The combination of AKI and sepsis is associated with more than 80% mortality [5]. This form of AKI has components of ischemia-reperfusion injury, direct inflammatory injury, coagulation and endothelial cell dysfunction, and apoptosis [6]. As sepsis-induced AKI has been associated with poor outcomes in hospitalized patients, we intended to study this form of AKI and compare it with AKI occurring in patients without sepsis for various clinical variables and outcomes.

## Materials and methods

This study was conducted in the department of nephrology at Sher-I-Kashmir Institute of Medical Sciences (SKIMS), Srinagar, a hospital in North India, over a period of two years with a total sample size of 200 patients selected randomly having sustained an acute kidney injury. This was a prospective, observational, and comparative study. We studied and compared two groups of patients with septic and non-septic AKI. Septic AKI patients included those who developed AKI after being diagnosed with sepsis (a life-threatening organ dysfunction caused by a dysregulated host response to infection).

Study population and inclusion criteria

All patients aged 16 years and more admitted to the department of nephrology and other departments of SKIMS with evidence of AKI were included in the study.

Exclusion criteria

Excluded in the study were patients with pre-existing end-stage renal disease (ESRD), those with age of <16 years, those who were treated with RRT before admission or for drug toxicity, and those who did not fulfill at least one predefined criterion for AKI defined as any of the following: 1) oliguria defined as urine output of <0.5 ml/kg/hour for >6 hours; 2) marked azotemia defined as serum urea of 30 mmole/L (84 mg/dl); 3) ≥1.5-fold rise in serum creatinine from the reference value, which is known or presumed to have occurred within one week; and/or 4) the need for acute renal replacement therapy (RRT).

The reference serum creatinine taken was the lowest creatinine value recorded within three months of the event. If a reference serum creatinine value was not available within three months, it was estimated from the nadir serum creatinine value if the patient recovered from AKI. Disease severity was assessed using the RIFLE classification [7]. The failure stage and onward were considered to be the severe form of AKI, while risk and injury classes were taken as mild and moderate forms of AKI.

Aims and objectives

The aim and objective of this study are as follows: 1) to study and compare the clinical profile of patients with septic and non-septic AKI in terms of age, gender, comorbidities, severity, etiology, and biochemical and sepsis-related parameters and 2) to study and compare the outcomes of patients with septic and non-septic AKI in terms of recovery of renal functions, duration of hospital stay, and mortality. The outcome of patients was studied in terms of mortality, days of hospital stay, and recovery of renal function at discharge with respect to RIFLE class, comorbidities, need for RRT, and etiology.

Statistical method

The data was first keyed into a Microsoft Excel (Microsoft® Corp., Redmond, WA) spreadsheet and cleaned for any inaccuracies. Statistical analysis was done using the Statistical Package for Social Sciences (SPSS) (version 27.0) (IBM SPSS Statistics, Armonk, NY). Categorical variables were shown in the form of frequencies and percentages. Also, Student's t-test and chi-square test were used to check the association between variables, and all results were discussed at 5% level of significance (p<0.05).

## Results

A total of 200 AKI cases were included in our study. Case distribution as per age and gender is depicted in Table 1. There was no discernible variation in the distribution of cases by gender, and cases were evenly distributed throughout the various age categories. The RIFLE classification was used to determine disease severity (Table 1).

**Table 1:** Case distribution across different variables AKI: acute kidney injury

Age groups	Total, n=200		Septic, n=80		Non-septic, n=120	
	Number of cases	%	Number of cases	%	Number of cases	%
18-40	66	33%	22	27.5%	44	36.7%
41-60	73	36.5%	31	38.7%	42	35%
≥61	61	30.5%	27	33.75%	34	28.4%
Gender						
Male	110	55%	32	40%	78	65%
Female	90	45%	48	60%	42	35%
AKI severity (RIFLE classification)						
R=risk	15	7.5%	05	6.25%	10	8.33%
I=injury	34	17%	09	11.25%	25	20.83%
F=failure	151	75.5%	66	82.5%	85	70.8%

The most frequent etiology in the non-septic group was AKI secondary to nephrotoxic exposure (27.5%), followed by acute glomerulonephritis (13.3%), vitamin D intoxication with hypercalcemia (12.5%), acute gastroenteritis (10.8%), and the combination of cardiorenal syndrome and post-cardiac arrest (10.0%) (Table 2).

**Table 2:** Etiology of non-septic AKI HRS, hepatorenal syndrome; AKI, acute kidney injury

Etiology	Number of cases	%
Nephrotoxins	33	27.5%
Acute glomerulonephritis	16	13.3%
Vitamin D intoxication with hypercalcemia	15	12.5%
Acute gastroenteritis	13	10.8%
Cardiorenal syndrome+post-cardiac arrest	12	10%
Postoperative	6	5%
Obstructive uropathy	5	4.16%
Acute pancreatitis	5	4.16%
Snake bite	5	4.16%
Rhabdomyolysis	3	2.5%
Multiple myeloma related	3	2.5%
Post stroke	2	1.66%
HRS	2	1.66%
Total	120	100%

In septic AKI, the most frequent etiology was urosepsis (37.5%), followed by chest sepsis (18.75%) (Table 3).

**Table 3:** Etiology in septic AKI AKI: acute kidney injury

Etiology and source of sepsis	Number of cases	%
Urine (urinary tract infections including pyelonephritis)	30	37.50%
Chest (community-acquired pneumonia {CAP} and aspiration pneumonia)	15	18.75%
Abdominal cavity (acute suppurative cholangitis and acute gastroenteritis)	11	13.75%
Genital tract (puerperal/postpartum sepsis)	8	10%
Skin and soft tissue (cellulitis and neutropenia with mucositis)	5	6.25%
CNS (meningoencephalitis)	1	1.25%
Occult/unknown	12	15%

Disease severity defined by the mean of maximum creatinine and urea was 5.230±3.382 and 125.11±73.88, respectively. The corresponding figures were significantly higher (5.881±3.435 and 146.61±74.25 ) in septic than in non-septic AKI cases (4.796±3.289 and 110.77±70.36) (p=0.027 and 0.001, respectively) (Table 4).

**Table 4:** Variables related to disease severity and outcome

Variable		N	Mean	Standard deviation	P value
Maximum creatinine	Total	200	5.230	3.382	0.027
	Septic	80	5.881	3.435	
	Non-septic	120	4.796	3.289	
Maximum urea	Total	200	125.11	73.88	0.001
	Septic	80	146.61	74.25	
	Non-septic	120	110.77	70.36	
Creatinine at discharge	Total	173	2.690	2.266	0.215
	Septic	58	3.126	2.660	
	Non-septic	115	2.470	2.016	
Improvement in creatinine	Total	173	59.06%	29.83%	0.168
	Septic	58	55.15%	28.93%	
	Non-septic	115	61.04%	30.21%	
Duration of hospital stay (days)	Total	200	8.465	5.828	<0.001
	Septic	80	10.713	7.012	
	Non-septic	120	6.967	4.302	

The mean duration of hospital stay was 8.465±5.828 days with 10.713±7.012 in the septic and 6.967±4.302 in the non-septic group of patients. The recovery of renal functions across different RIFLE groups as per percentage improvement in creatinine is depicted in Table 5.

**Table 5:** Recovery of renal functions

RIFLE class	Percentage improvement in creatinine				P value
	Septic		Non-septic		
	Mean	Standard deviation	Mean	Standard deviation	
Risk and injury	72.69%±22.08%		82.51%±19.28%		0.188
Failure	50.57%±28.94%		51.64%±29.3%		0.843
P value	0.009		<0.001		

Patients in the failure class of RIFLE had longer (9.391±6.340) duration of hospital stay compared to patients in the injury group (5.837±2.511 days) (p<0.001) (Table 6). The overall mortality in AKI patient was 13.5% (27 cases); mortality in the septic group was higher (27.5%) than in the non-septic group (4.16%).

**Table 6:** Duration of hospital stay SD: standard deviation

RIFLE class	Total	Septic	Non-septic	P value
	Mean±SD	Mean±SD	Mean±SD	
Risk and injury	5.837±2.511	7.571±3.480	5.143±1.593	0.024
Failure	9.391±6.340	11.379±7.400	7.847±4.888	0.001
P value	<0.001	0.006	<0.001	

## Discussion

A total of 200 cases of AKI were enrolled. Cases were evenly distributed along different age groups, and there was no significant difference in gender distribution. Disease severity was defined using the RIFLE classification. There was no significant difference in numbers based on the RIFLE class distribution between septic and non-septic AKI groups (p=0.060), though the maximum patients identified belonged to the failure class, which is consistent with the studies on AKI by Cruz et al. [8] and Hoste et al. [9].

In the septic AKI, 30 (37.5%) patients had urosepsis (sepsis due to urinary tract infections including pyelonephritis), followed by chest sepsis (due to respiratory tract infections including community-acquired pneumonia {CAP} and aspiration pneumonia) in 15 (18.75%) patients and sepsis due to abdominal cavity infections (acute suppurative cholangitis and acute gastroenteritis) in 11 (13.75%) patients. Puerperal/postpartum sepsis was present in eight (10%) patients. Less common etiologies were pyelonephritis, skin and soft tissue infections, and meningoencephalitis. Bagshaw et al. [10] recognized the chest as the commonest source of sepsis, followed by the abdomen. AKI secondary to nephrotoxic exposure was the commonest cause in the non-septic group, in 33 (27.5%) patients. Other common etiologies were acute glomerulonephritis in 16 (13.3%) of the cases, vitamin D intoxication with hypercalcemia in 15 (12.5%), acute gastroenteritis in 13 (10.8%), and the combination of cardiorenal syndrome and post-cardiac arrest in 12 (10%). Postoperative AKI was found in six (5%) of the cases, whereas snake bite, acute pancreatitis, and obstructive uropathy were found to be the causes in five (4.16%) of the cases. Rhabdomyolysis was found in three (2.5%) of the cases and so was found in multiple myeloma-related AKI. Lastly, AKI complicated two (1.66%) cases, each with stroke and hepatorenal syndrome. Zappitelli [11], in his study, found sepsis, nephrotoxic medication, and ischemia as the most common causes of AKI.

The literature across many parts of the globe has shown that patients of septic AKI suffer greater derangements in their physiological markers and laboratory parameters along with a higher burden of illness and more severe degrees of AKI. Similar findings were seen in our study, e.g., as seen by Bagshaw et al. [10]. Disease severity defined by the mean of maximum creatinine and urea was 5.230±3.382 and 125.11±73.88, respectively. The corresponding figures were significantly higher (5.881±3.435 and 146.61±74.25 ) in septic than in non-septic AKI cases (4.796±3.289 and 110.77±70.36) (p=0.027 and 0.001, respectively). Of the patients that survived the insult of AKI, the mean creatinine at discharge was 2.690±2.266 and the percentage improvement in creatinine at discharge was 59.06%±29.83%. There were no significant differences in the mean creatinine at discharge between septic and non-septic AKI groups (p=0.215) and the percentage improvement in the creatinine in the same groups (p=0.168). This is consistent with the studies of Bagshaw et al. [10] and Cruz et al. [12]. The mean duration of hospital stay was 8.465±5.828 days with 10.713±7.012 in the septic and 6.967±4.302 in the non-septic group of patients. It was longer and statistically significant in patients with septic AKI (p<0.001). This is consistent with the study of Hamzic-Mehmedbasic et al. [13] who found that the mean hospital stay prior to the RRT commencement was significantly longer in septic AKI patients when compared to non-septic AKI patients (10.6 days versus two days, p=0.03). Barrantes et al. [14] found that the median of the duration of hospital stay for AKI patients was seven days. Bagshaw et al. [10] also found longer duration of hospital stay in septic AKI patients than in non-septic AKI groups. Patients in the failure class of RIFLE had longer (9.391±6.340) duration of hospital stay compared to patients in the risk and injury groups (5.837±2.511 days, p<0.001). The same was true regarding RIFLE classes irrespective of septic and non-septic etiologies. Bagshaw et al. [15] found that the median length of stay increased from 3.1 to 4.8 days as septic AKI patients progressed from RIFLE-injury to RIFLE-failure class, which is consistent with our study.

The overall mortality in AKI patient was 13.5% (27 cases), which is similar to the study by Barrantes et al. [14] who found mortality of 14.8% in the non-critically ill patients of AKI. The mortality in the septic group was higher (27.5%) and statistically significant (p<0.001) than in the non-septic group (4.16%). It has been established in numerous studies that the septic AKI carries higher mortality; e.g., Hamzic-Mehmedbasic et al. [13] also found in their study that septic AKI patients had significantly higher in-hospital mortality in comparison to non-septic AKI patients (14.7% versus 3.03%, p=0.04). Mehta et al. [16] found lower numbers: 48% for patients with sepsis before AKI and 44% for patients with sepsis after AKI. The in-hospital mortality of patients with septic AKI reported by Neveu et al. [17] was 75%. There was no significant effect of gender on mortality (p=0.495). Likewise, there were no significant differences in the mortality between males and females within septic and non-septic groups (p=0.995 and 0.972, respectively).

A total of 32 patients (16%) required RRT in the form of either hemodialysis or peritoneal dialysis, out of which nine patients expired (mortality: 28.125%). Among the patients who did not require dialysis support, mortality was 10.71%. Patients requiring renal replacement therapy (RRT) had higher mortality, which was statistically significant than the patients not requiring RRT (p=0.040). This is consistent with the study by Choi et al. [18], Prakash et al. [19], and Liaño and Pascual [20] who found greater mortality in the patients who had received dialysis. There was no significant difference in the mortality between the septic and non-septic AKI patients as far as the need for RRT is concerned (p=0.538).

The disease severity (defined by RIFLE class) had significant effect on the mortality and other outcomes in our study. The mortality in the failure class was higher (16.55%) than that in the injury (4.08%) and risk (0%) classes (p=0.026). This is consistent with the study by Cruz et al. [8], Bagshaw et al. [15], and Hoste et al. [9]. Uchino et al. [21] reported that there was an almost linear increase in the hospital mortality rate with increasing RIFLE class. Also, the mortality in the septic AKI patients in the failure group was significantly higher than the non-septic patients (30.30% versus 5.88%, p<0.001) in the same class, an effect of sepsis as discussed above. This is consistent with the study by Bagshaw et al. [15] who found that septic AKI was associated with greater severity of AKI across all RIFLE strata compared to non-septic AKI. The same is true regarding mortality in injury class among septic and non-septic patients (14.28% versus 0%), but this difference is not statistically significant (p=0.074), likely due to the small sample size available in this subgroup of patients. There was no significant effect on mortality in AKI of the risk factors such as the following: 1) diabetes mellitus (mortality of 15.1% in those with diabetes versus 13.1% in those without diabetes, p=0.955); 2) cerebrovascular accident (CVA) (mortality of 20% in those with CVA versus 13.3% in those without CVA, p=0.911), which is consistent with the previous studies by Ostermann and Chang [22], Prakash et al. [19], and Cruz et al. [8] who found no effect of diabetes and cerebrovascular accident on mortality in AKI; 3) malignancy (mortality of 21.24% versus 12.9% in those with and without malignancy, respectively; p=0.667); 4) chronic kidney disease (CKD) (mortality of 14.2% versus 13.4% in those with and without CKD, respectively; p=0.997); 5) chronic liver disease (CLD) (mortality of 33.3% versus 13.2% in those with and without CLD, respectively; p=0.599), which is inconsistent with a previous study by Ostermann and Chang [22], in which the reasons may be smaller sample size, single-center study, and the effect of other risk factors on outcome; and 6) exposure to nephrotoxic drugs (mortality of 0%).

On the other hand, the following comorbidities were found to have significant effect on mortality among the AKI patients: 1) sepsis (mortality of 27.5% versus 4.16% in those with and without sepsis, respectively; p<0.001), consistent with the study by Mehta et al. [16]; 2) acute coronary syndrome (ACS) (mortality of 33% versus 12% in those with and without ACS, respectively; p=0.029), consistent with the studies by Gallagher et al. [23] and Pereira et al. [24]; 3) the need for vasopressor support (mortality of 57.14% versus 8.37% in those with and without the need for vasopressors, respectively; p<0.001), which is consistent with the study by Uchino et al. [21]; 4) multiorgan dysfunction syndrome (MODS) (mortality of 44.11% versus 7.13% in those with and without MODS, respectively; p<0.001), which is consistent with the study by Ostermann and Chang [22]; 5) age of >65 years (mortality of 28.5% versus 10.3% in those above and below 65 years of age, respectively; p=0.016), which is consistent with a previous study by Ostermann and Chang [22]; 6) mechanical ventilation (mortality of 50% versus 13.13% in those with and without the need for mechanical ventilation, respectively; p=0.025), which is consistent with the studies by Uchino et al. [21], Ostermann and Chang [22], and Prakash et al. [19]; and 7) septic shock (mortality of 80% versus 10% in those with and without septic shock, respectively; p<0.001), which is consistent with the studies by Uchino et al. [21], Bagshaw et al. [10], and Prakash et al. [19].

Limitation

The study did not include patients who were admitted for various surgical procedures and developed complications with AKI, which could be a limitation of the study. Additionally, the sample size was small, and the study was conducted at a single center, which may limit the generalizability of the findings.

## Conclusions

The most prevalent cause of AKI in the septic group was urosepsis, whereas the most common cause in the non-septic group was nephrotoxin exposure. Compared to patients with non-septic AKI, patients with septic AKI had significantly longer hospital stays (10.71±7.012 days in the septic group compared to 6.967±4.302 days in the non-septic group) and higher in-hospital mortality rates (27.5% in the septic group as compared to 4.16% in the non-septic group). Sepsis had little effect on renal functions, as seen by the urea and creatinine levels at discharge. Mortality was significantly affected by the age of over 65, the requirement for mechanical ventilation, the use of vasopressors and RRT, and the presence of MODS, septic shock, and ACS.
